# Efficacy of Diosmectite (Smecta)^®^ in the Treatment of Acute Watery Diarrhoea in Adults: A Multicentre, Randomized, Double-Blind, Placebo-Controlled, Parallel Group Study

**DOI:** 10.1155/2011/783196

**Published:** 2011-06-30

**Authors:** Faouzi Khediri, Abdennebi Ilhem Mrad, Moussadek Azzouz, Hedi Doughi, Taoufik Najjar, Hélène Mathiex-Fortunet, Philippe Garnier, Antoine Cortot

**Affiliations:** ^1^83 avenue Mohamed V, 1002 Tunis, Tunisia; ^2^Regional Hospital, Route Mornag—Yasminet, 2013 Ben Arous, Tunisia; ^3^Tahar Mamouri Regional Hospital, 8000 Nabeul, Tunisia; ^4^Interior Security Forces Hospital, 2070 La Marsa, Tunisia; ^5^Charles Nicolle Hospital, Boulevard du 9 avril, 1006 Tunis, Tunisia; ^6^Ipsen, 65 quai Georges Gorse, 92100 Boulogne-Billancourt, France; ^7^Univ Lille Nord de France, F-59000 Lille, France; ^8^CHU Lille, F 59000 Lille, France

## Abstract

*Background*. Although diosmectite has demonstrated efficacy in the treatment of acute watery diarrhoea in children, its efficacy in adults still needs to be assessed. The objective of this study was therefore to assess the efficacy of diosmectite on the time to recovery in adults with acute diarrhoea. *Methods*. A total of 346 adults with at least three watery stools per day over a period of less than 48 hours were prospectively randomized to diosmectite (6 g tid) or placebo during four days. The primary endpoint was time to diarrhoea recovery. *Results*. In the intention-to-treat population, median time to recovery was 53.8 hours (range [3.7–167.3]) with diosmectite (*n* = 166) versus 69.0 hours [2.2–165.2] with placebo, (*n* = 163; *P* = .029), which corresponds to a difference of 15.2 hours. Diosmectite was well tolerated. *Conclusion*. Diosmectite at 6 g tid was well tolerated and reduced the time to recovery of acute watery diarrhoea episode in a clinically relevant manner.

## 1. Introduction

Acute diarrhoea is one of the leading causes of morbidity worldwide [1]. The annual rate of diarrhoea among adults in Western Europe and the United States averages about one episode per person per year [2–4]. Episodes are usually brief, not life-threatening and most often self-limited but symptoms can be disturbing and incapacitating. Urgency, loose stools, abdominal discomfort, and inconvenience such as loss of faecal continence make it an unpleasant and distressing condition. It is commonly recognized that these symptoms lead to substantial costs for society as it is estimated that half of the episodes are related to missed workdays [5, 6].

Various guidelines are available for the treatment of acute diarrhoea in adults [6–8]. Fluid intake is to be maintained, preferably with glucose-containing drinks or electrolyte-rich soup, as indicated by thirst. Oral rehydration solutions are needed in frail people only. Small light meals can be recommended, solid food intake being guided by appetite. Several treatment options are available: antidiarrhoeal therapies such as antimotility, anticholinergic, antisecretory, and antimicrobial drugs, as well as adsorbents.

Diosmectite, an activated natural aluminosilicate clay consisting of a double aluminium and magnesium silicate, is an adsorbent widely used for the treatment of acute infectious diarrhoea in children. In children, diosmectite efficacy in the treatment of acute watery diarrhoea has been assessed in a recent meta-analysis. Combined data from six randomized, controlled trials have shown that diosmectite significantly reduces diarrhoea duration by one day and increases the chance of recovery on intervention day three versus control group [9]. Two recent trials have indicated that diosmectite reduces stool output (versus placebo) in children with acute watery diarrhoea [10].

In adults, no prospective, randomized, placebo-controlled trial on the efficacy of diosmectite in the treatment of acute diarrhoea has yet been conducted. Diosmectite was compared to loperamide only, in four open, prospective trials that showed similar effects for both drugs in the treatment of acute infectious diarrhoea [11–14]. The clinical effect of diosmectite has nonetheless been studied in functional diarrhoea [15, 16], radiation-induced diarrhoea [17], irinotecan-induced diarrhoea [18], and AIDS-associated chronic idiopathic diarrhoea [19].

Like other adsorbents, diosmectite is not absorbed in the intestine. It can adsorb eight times its own weight of water, thereby diminishing free stool water. It also adsorbs toxins, bacteria, and rotavirus, preventing their adherence to intestinal membranes. Diosmectite strengthens the mucosal barrier, and, in the absence of mucus, prevents its disruption [20–22]. By consequence, on the contrary to some antidiarrhoeal agents acting on motility, diosmectite could decrease the time infectious agents remaining in the intestine. However the absorbing characteristics of diosmectite can disturb the absorption rates of other substances. Therefore, the concomitant use of other medicinal products is not recommended. Furthermore, this pharmacological profile is accompanied by a good safety profile [9].

This demonstrated efficacy in children suggests that, compared to placebo, diosmectite could improve recovery from acute watery diarrhoea in adults, but this has never been studied. We therefore undertook a multicentre, randomized, double-blind, placebo-controlled trial to assess the efficacy of diosmectite for the treatment of acute watery diarrhoea in adults.

## 2. Materials and Methods

### 2.1. Patients

The study included outpatient males and nonpregnant women aged 18 to 65 years in 23 primary and secondary care centres in Tunisia.

Inclusion criteria were an acute diarrhoea episode defined as at least three watery stools per day over a period of 48 hours or less, and patients with usually normal bowel movements, that is, at least three normal stools per week and three or less normal stools per day.

Exclusion criteria related to the diarrhoea episode were fever >39°C, blood or pus in stools, previous history of acute watery diarrhoea over the past 30 days, dehydration requiring intravenous rehydration, traveller's diarrhoea, history of chronic diarrhoea (three or more loose or watery stools per day for at least 12 weeks, consecutive or not, in the preceding 12 months), and motor diarrhoea defined as urgent, morning postprandial need for defaecation. Exclusion criteria related to drug use were patients having used antidiarrhoeal agents over the month prior to baseline, patients requiring the daily intake of a drug treatment with narrow therapeutic margin, and patients with diarrhoea possibly induced by antibiotics, laxative agents, thyroid hormones, or colchicine.

### 2.2. Study Design

This was a multicentre, placebo-controlled, double-blind, randomized study with parallel groups conducted in 23 centres in Tunisia. Tunisia was chosen for its good medical practice and compliant organization and its prevalence of acute infectious diarrhoea comparable to that of industrialized countries [4, 23].

Newly diagnosed ambulatory patients suffering from acute diarrhoea presumed to be of infectious origin were randomized to receive diosmectite or placebo. During their participation, patients recorded in diaries their stool frequency, presence of blood in stools, and abdominal pain/cramps. An acute diarrhoea episode was regarded to have resolved after the patient had one formed stool followed by a nonwatery stool.

The study was registered at http://www.clinicaltrials.gov/ under the reference NCT00276328. The study was conducted in accordance with the declaration of Helsinki (Somerset West, Republic of South Africa, October 1996), with the Tunisian regulatory texts relative to the protection of persons participating in biomedical research, and with the applicable Good Clinical Practices requirements (USFDA 21CFR-1A part 50 subpart D concerning children in clinical investigations; European Clinical Trials Directives 2001/20/EC and 2005/28/EC, corresponding to ICH E6). The Independent Ethics Committee of Tunis, Tunisia, approved the study protocol in January 2005. Patients gave their informed consent before inclusion.

### 2.3. Treatment

Patients were randomly treated with either diosmectite or placebo. Patients were randomized at visit 1, in sequential ascending order within each centre. The investigator only dispensed the study drug to the patients included in the study. For each study site, the sponsor-assigned biostatistician prepared a list of treatment allocation codes, which were confidentially supplied to the drug supplier. The master list and the copy given to the Clinical Trial Supplies Unit were kept confidential in a safe and secure location. The randomization list was not released until approval was received for the study to be unblinded for analysis. For both diosmectite and placebo, treatment was two sachets each containing 3 g of powder for oral suspension three times a day (morning, lunch and dinner). From day 1 to day 4, the treatment was mandatory (i.e., six sachets per day). From day 5, the dose was six sachets per day until recovery, that is, one formed stool followed by a nonwatery stool, with a maximum of seven days of treatment.

Diosmectite is a powder for oral suspension in a sachet, composed of 3.000 g diosmectite, 0.004 g vanillin, 0.007 g sodium saccharin, and 0.749 g glucose monohydrate. A placebo formula was specifically developed. It was a powder for oral suspension in a sachet, composed of 1.000 g titanium dioxide, 1.181 g maltodextrin (Roquette Glucidex IT 38), 0.004 g vanillin, 0.007 g sodium saccharin, 2.150 g glucose monohydrate, and 0.018 g caramel colouring E150B. Placebo was inert and identical to diosmectite in size, weight, colour, smell, taste, and appearance, either as a powder or a water solution. Its absence of pharmacological activity was demonstrated on an animal model of watery diarrhoea (data not shown). Treatment compliance was assessed at visit 2 or 3, based on sachet consumption recorded in the patient diary, overall count of used and unused sachets, and answers to questions on study drug compliance.

### 2.4. Procedures

Patients attended the study centres three times: at screening (patients included in the study began treatment at once), at midstudy (day 4 or 5 after inclusion), and for a concluding examination (day 8 or 9 after inclusion).

At baseline visit (visit 1), written informed consent was collected, patients were given a diary, and the following data were collected: demographics, vital signs, weight, physical examination results, use of concomitant medication, previous medical history, and case history of the acute diarrhoea episode including date of first watery stool, number of stools over the past 24 hours, and presence of other associated symptoms over the past 24 hours (nausea, abdominal pain, anal irritation). Patients were asked to record the following data every day in the diary: date, hour of stool onset, stool consistency (watery, loose, formed, or hard), presence of symptoms such as nausea, abdominal pain, and anal irritation, and study drug consumption (number of sachets taken each day). To standardize the rating of stool consistency, patients were shown a scheme explaining the different stool consistencies and corresponding ratings [24]. A stool was sampled for microbiological and parasitic examination at baseline.

During the second and the third visits (visit 2 and visit 3), investigators collected vital signs, physical examination, weight, adverse events, clinical data, study drug use in the diary, stool consistency, and stool time. In addition, the number of treatment sachets used and unused that were kept by the investigator was recorded at visit 3. 

### 2.5. Objectives

The primary objective was to compare the efficacy of diosmectite to that of placebo in adults with acute watery diarrhoea, taking time to recovery as a primary endpoint. The secondary objectives were to compare diosmectite and placebo with regards to the other efficacy parameters and safety in adults with acute watery diarrhoea.

### 2.6. Primary Outcome Measure

Time to recovery was defined as the time (hours) from first study drug intake (H0) to diarrhoea recovery. Recovery was defined as the first formed or hard stool followed by a nonwatery stool. Time to recovery was determined from the data collected in diaries. However, if the diary was lost or unusable, analyses were performed from data collected in the case report form, after blind review decision.

### 2.7. Secondary Outcome Measures

Secondary efficacy endpoints were time (hours) from the first sachet intake to the last watery stool and, per 12-hour period, number of stools, number of watery stools, percentage of patients having recovered (defined as having achieved the primary efficacy endpoint), and percentage of patients with associated symptoms such as nausea, abdominal pain, and anal irritation.

### 2.8. Tolerability

The safety evaluation was carried out during the follow-up visits and was based on monitoring of any adverse event (AE) occurring from the moment patients had given informed consent to 7 days after the end of the study. AEs were coded according to the Medical Dictionary for Regulatory Activities (MedDRA) version 9.1. Safety variables were the frequency of adverse events, with a special attention to incidence of nausea, abdominal pain, and anal irritation. 

### 2.9. Statistics

Primary and secondary endpoints were compared in both groups using appropriate statistical tests: Wilcoxon's test for quantitative parameters without normal distribution described by median and range; Student's *t*-test for quantitative parameters with normal distribution described by mean and standard deviation; Mantel-Haenszel's test, Chi-square or Fisher's test for qualitative parameters described by frequency and percentage.

With regards to the primary endpoint, statistical analysis was based on Wilcoxon's test in the intention-to-treat (ITT) population. The ITT population included randomized patients having taken the study drug at least once together with a primary endpoint that was assessable. Per-protocol (PP) population included ITT patients without major protocol deviations as defined after a blind review. PP analyses were supportive only. To assess robustness of the results, it was decided to perform post hoc analyses of primary efficacy data in ITT and PP populations using the “time to event” Gehan-Wilcoxon test, which takes into account censored data and their specific distributions with early events and late censures. Secondary efficacy analyses were conducted in the ITT population.

Sample size determination was based on the hypothesis that time to recovery was significantly shorter under diosmectite than under placebo (one-sided hypothesis). From previous studies, the expected difference of the primary efficacy criterion between diosmectite and placebo was 24 hours, with an estimated standard deviation (SD) of 61.7 hours. With an alpha risk of 5% and a beta risk of 20%, the number of patients to be included per group was 140 to obtain 104 “assessable” patients per group, that is, for which the primary outcome could be assessed. 

Statistical analyses were performed using SAS (SAS Institute, version 8.1, North Carolina, USA).

## 3. Results

### 3.1. Patient Disposition and Characteristics

**Figure 1:**
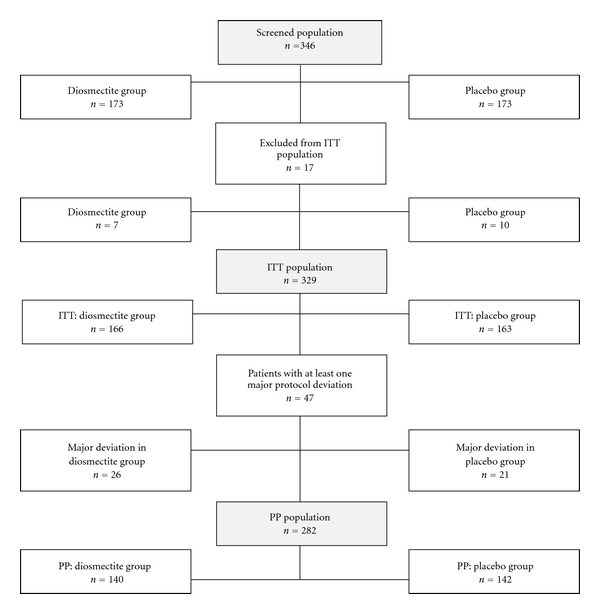
Flow chart of study populations. Diosmectite (6 g three times a day) or placebo in the treatment of acute diarrhoea in adults.

Between January 2005 and July 2006, 23 physicians assessed 346 patients for eligibility (from 1 to 52 patients per physician, mean = 15 patients per physician). A flow chart of all the screened patients (*n* = 346) included and randomized to receive diosmectite (*n* = 173) or placebo (*n* = 173) is shown in Figure 1. The total of the 346 included/randomized patients was evaluated for safety. A total of 329 patients constituted the ITT population. The 17 patients excluded from the 346 screened patients for ITT analysis had been invalidated during the blinded review following an on-site audit, which revealed that they might not have fully completed their diary.

Major protocol deviations were observed in 47 patients: 26 patients (15.7%) in the diosmectite group and 21 patients (12.9%) in the placebo group. These 47 patients were excluded from ITT population to constitute the PP population (*n* = 282). The most frequent deviations in both groups were insufficient stool recording in the diary making it impossible to calculate the time to recovery (*n* = 39 deviations), lack of stool recording or of date/time of recovery (*n* = 34), poor compliance to the treatment (*n* = 14), or previous or concomitant use of forbidden treatment (*n* = 5). Of the 47 patients with major protocol deviation, 20 were patients that had not recovered by the end of the study period (i.e., seven days). In contrast, another 5 of these 47 patients had major protocol deviations due to poor observance as a consequence of early recovery.

The two treatment groups were well balanced for basic demographic data and disease history. There was no difference between groups with regards to age, gender distribution, height, and weight, previous medical history (222/329 or 67.5% of the patients had no medical history) and concomitant treatments. The baseline clinical picture of acute diarrhoea episodes was not different between both groups (Table 1).

### 3.2. Primary Efficacy Evaluation

In the ITT population (*n* = 329), the median [range] time to recovery was significantly shorter in the diosmectite group (53.8 hours [3.7–167.3]) than in the placebo group (69.0 hours [2.0–165.2]) when tested with Wilcoxon's test (*P* = .0294). The difference between the two groups was 15.2 hours. The statistical significance of this effect was also evidenced by the post hoc analysis using the time-to-event Gehan-Wilcoxon test considering censored data: 56.3 hours [47.7– 68.0] in the diosmectite group *versus* 72.2 [63.3– 82.0] hours in the placebo group (*P* = .0291).

In the PP analysis (*n* = 282), median time to recovery was 54.5 hours [3.7–167.3] for the diosmectite group and 68.2 hours [2.2–165.0] for the placebo group when tested with Wilcoxon's test (*P* = .067). The difference between the median times to the end of the acute watery diarrhoea episode was 13.7 hours. In accordance with the protocol, the PP population excluded patients with major protocol deviations including patients who recovered early and, as a consequence, prematurely stopped their treatment. Five of these patients had prematurely stopped treatment because of a perfectly documented recovery. When including these patients in a post hoc analysis, the Gehan-Wilcoxon time to event test confirmed the statistical significance of the difference, in favour of diosmectite (*P* = .039). 

### 3.3. Secondary Efficacy Evaluation

**Figure 2:**
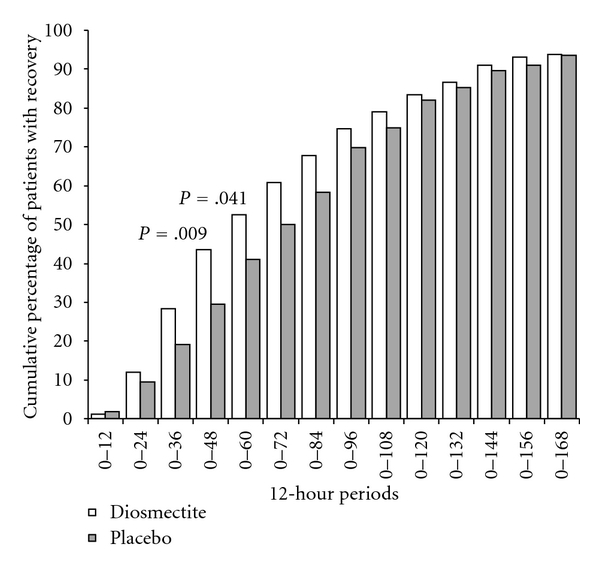
Cumulative percentages of recovered patients per 12 h period. Recovery was the first formed stool followed by a nonwatery stool (primary endpoint). Diosmectite (6 g three times a day) or placebo in the treatment of acute diarrhoea in adults.

The percentage of therapeutic success, defined as patients having achieved the primary efficacy endpoint, per cumulative 12-hour period, was higher in the diosmectite group than in the placebo group in the following periods: 0–36 h (28.5% versus 19.2% [*P* = .055]); 0–48 h (43.7% versus 29.5% [*P* = .009]); 0–60 h (52.5% versus 41.0% [*P* = .041]); 0–72 h; 60.8% versus 50.0% [*P* = .055]) (Figure 2). 

Median [range] time from first sachet intake to the last watery stool was 20.5 hours [0.0–160.8] in the diosmectite group and 23.0 hours [0.0–223.8] in the placebo group (*P* = .569). The median number of stools per 12-hour period decreased from 3 at baseline to 1 at the 36–48 h period onwards with a significant difference in favour of diosmectite group at the 72–84 h period (*P* = .016). The median number of watery stools decreased from 2 at baseline to 0 at the 12–24 h period onwards (N.S). 

Nausea during the 24 hours before inclusion was present in 71.7% of the patients in the diosmectite group and 68.7% in the placebo group. Incidence decreased dramatically to <5% in both groups after 48 hours (N.S).

 Abdominal pain before inclusion was present in 86.1% of the patients in the diosmectite group and 78.5% in the placebo group. Incidence decreased to <15% in both groups after 48 hours (N.S). Anal irritation before inclusion was present in 18.1% of the patients in the diosmectite group and 24.5% in the placebo group. Incidence decreased to <5% in both groups after 36 hours (N.S).

### 3.4. Safety and Tolerability

Both diosmectite and placebo were well tolerated. The median [range] duration of exposure was 4.2 days [0.3–7.5] in the diosmectite group and 4.2 days [0–10.1] in the placebo group. In total, 12 AEs occurred in 11 patients during the study: 6 AEs in 6 patients (3.5%) of the diosmectite group and 6 AEs in 5 patients (2.9%) of the placebo group. In both groups, the most frequently reported AEs were gastrointestinal disorders.

Particularly, incidence of new nausea episodes during the study was observed in 4.2% (7/166) of the patients in the diosmectite group and 3.7% (6/163) in the placebo group. Incidence of abdominal pain episodes was observed in 2.4% (4/166) of the patients in the diosmectite group and 8.6% (14/163) in the placebo group. Finally, incidence of anal irritation episodes was observed in 12.6% (21/166) of the patients in the diosmectite group and 29.4% (48/163) in the placebo group. 

Two serious AEs were reported in two patients in the placebo group: one case of fracture of the lower limb and one case of appendicitis; both were assessed as unrelated to the study drug. AEs leading to permanent study medication discontinuation were reported in 3 patients in the diosmectite group (1.7%) and in 3 patients in the placebo group (1.7%). Gastrointestinal disorders (constipation, abdominal pain, appendicitis, and amoebiasis) were the main reason for discontinuation due to AEs in both groups.

During the study, no relevant abnormality was found with regards to body weight, blood pressure, and cardiac rhythm.

## 4. Discussion

This is the first randomized, placebo-controlled trial prospectively comparing diosmectite to placebo for the treatment of acute diarrhoea in adults. This study showed that oral diosmectite sachet 6 g three times a day significantly shortened time to recovery in the treatment of acute diarrhoea in adults. This was further supported by the results found in the PP population. This study also confirmed the good safety profile of diosmectite, as illustrated by the limited number of AEs, of which only 3 were considered drug related (constipation).

The statistical analysis plan was based upon the assumption that the duration of the diarrhoea episode would be shorter than seven days for all patients, without any risk of data censure. It was therefore planned to compare mean diarrhoea durations using the Wilcoxon's test, which is perfectly adapted to this type of data. The definition of diarrhoea duration required that patients are followed after the first formed stool to confirm the end of the diarrhoea episode. This definition of recovery was selected to guarantee the clinical relevance of the primary criterion. Of note is that it was much more constraining than previous trials, which defined recovery as the first nonliquid stool. However, according to the definition of recovery used in the study, 35 patients showed diarrhoea duration longer than seven days. Since the protocol planned a seven-day followup, these patients were censored in statistical analyses. Nevertheless, a post hoc time to event analysis taking data censure into account was carried out. The Gehan-Wilcoxon test was preferred to the Logrank test because of the particular distribution of the events considered and the onset of censures during study followup. Indeed, the Gehan-Wilcoxon test is more adapted than the Logrank test to early events and late censures. Moreover the latter is based upon the assumption of proportional hazards, which is most probably not verified in this trial since the active treatment is supposed to shorten time to recovery without modifying the risk of recovery. Acute watery diarrhoea is self-resolving, even in the absence of treatment. The results of the Gehan-Wilcoxon test confirmed the effectiveness of diosmectite. These results are consistent with the primary analysis and confirm that diosmectite shortens time to recovery.

Despite significantly shorter time to recovery in the diosmectite group, the proportions of patients achieving recovery were similar in both groups at the end of the study. This is explained by acute watery diarrhoea being self-resolving within seven days.

The trial was performed in a homogeneous Tunisian population with positive stool culture in 26% of the patients. These figures are consistent with those reported in the literature and previously in Tunisia [4, 6, 23, 25]. Of note is that most patients had a recent episode of acute diarrhoea, similar in both groups (median time from first watery stool to treatment onset = 1 [0–3] day from the 1st watery stool to inclusion (NS)), with at least one associated symptom such as nausea, abdominal pain, or anal irritation in >90% of the patients and a median number of six stools per day before treatment onset. Hence, it can be extrapolated that if the primary endpoint variable had been measured from the time of onset of diarrhoea, instead of from the first intake of study drug, the difference between the two groups would still have been the same, that is, 15.2 hours. Moreover, this pattern of diarrhoea is in accordance with the definition of acute diarrhoea in developed countries [4, 25, 26]. Therefore, it can be estimated that results of the present study can be extrapolated to western countries.

The endpoints most frequently used in trials regarding antidiarrhoeal drugs in children and adults are stool volume and time from treatment onset to last liquid or first formed stool [12, 14, 26, 27]. Except in chronic diarrhoea, trials performed in adults rarely use stool volume as an endpoint. The clinical effect of diosmectite as an antidiarrhoeal agent in adults has been assessed mainly by the measurement of time to transit normalization [6, 26]. The definition of recovery chosen is again more stringent since it is based not only on the achievement of a normal stool but also by its following a nonwatery stool, thereby reflecting an actual cessation of the acute diarrhoea episode. 

The only data to which the present results may be compared derive from trials comparing diosmectite to loperamide in the treatment of acute diarrhoea in adults [11–14]. However, heterogeneity in trial design, drug doses, and endpoint definition makes these results difficult to compare with those presented here. It can only be inferred from these studies that, depending on the modalities of treatment and recovery definition, diosmectite and loperamide can show similar improvements of the duration of acute diarrhoea in adults. This is further supported by the results from the prospective trials comparing loperamide to placebo in acute diarrhoea in adults [27–30]. In one study the endpoint was the mean number of stools per day [30] but in the other three studies, the definition of time to recovery was not very different to that chosen here: time between the first drug intake and the first 24-hour period without watery or loose stool that was not followed by the recurrence of diarrhoea during the following 24–48 hours. In these three trials, median times to recovery were respectively: 45 hours 15 minutes in the placebo group versus 23 hours and 30 minutes in the 1 mg loperamide group [27]; 34 hours 15 minutes in the placebo group versus 26 hours 30 minutes in the 1 mg loperamide group [29]; 40 hours 35 minutes in the placebo group versus 27 hours 55 minutes in the 1 mg loperamide group [28]. This corresponds to respective decreases of 21 hours, 12 hours 40 minutes, and 7 hours 45 minutes with loperamide, which can be considered to be a similar range to the results observed here with diosmectite. In addition, the trial data presented here employed a more stringent definition of recovery. In studies comparing loperamide to placebo, time to recovery was time to the last watery stool whereas in the present study it was time to the first formed or hard stool followed by a nonwatery stool.

## 5. Conclusions

This randomized, double-blind, placebo-controlled trial shows that diosmectite at a dose of 6 g three times a day reduced the time to recovery of an acute watery diarrhoea episode in adults. Diosmectite was also associated with a very good safety profile and did not decrease intestinal peristalsis. In summary, the results of the present study support the use of diosmectite in the management of acute watery diarrhoea in adults.

## Figures and Tables

**Table 1:** Demographics, previous medical history, and characteristics of the acute diarrhoea episode.

	Diosmectite	Placebo	*P* value
	*N* = 166	*N* = 163	
Demographics			
Male, *n* (%)	89 (53.6)	86 (52.8)	.88^a^
Age (years), median [range]	38.0 [19–63]	38.0 [19–66]	.84^b^
Height, females (cm), median [range]	162.0 [150–180]	162.0 [150–182]	.74^b^
Height, males (cm), median [range]	172.0 [158–202]	173.0 [158–189]	.33^b^
Weight, females (kg), median [range]	64.0 [40–94]	66.0 [44–102]	.29^b^
Weight, males (kg), median [range]	78.0 [49–107]	76.0 [55–152]	.24^b^

Characteristics of the diarrhoea episode			
Previous history of gastrointestinal disorders, *n* (%)	9 (5.4)	13 (8.0)	.35^a^
Days from the 1st watery stool to inclusion, median [range]	1.0 [0–3]	1.0 [0–3]	.90^b^
Nausea, abdominal pain, or anal irritation, *n* (%)	156 (94)	148 (90.8)	.28^a^
Number of stools over the past 24 hours, median [range]	5.0 [2–22]	6.0 [3–20]	.17^b^
Positive stool culture, *n* (%)	40 (29.2)	33 (22.9)	.23^a^
Rotavirus	16 (11.9)	14 (10.0)	.61^a^
Adenovirus	7 (5.4)	3 (2.2)	.21^a^
*Escherichia coli *	13 (9.6)	13 (9.3)	.92^a^
*Staphylococcus aureus *	4 (2.9)	2 (1.4)	.44^a^
Amoebiasis	4 (2.9)	1 (0.7)	.21^a^

^a^2-tailed Chi-square test; ^b^Wilcoxon's test.
